# Prevalence and associated risk factors of anemia among HIV infected children attending Gondar university hospital, Northwest Ethiopia: a cross sectional study

**DOI:** 10.1186/s12878-015-0032-6

**Published:** 2015-09-24

**Authors:** Bamlaku Enawgaw, Meseret Alem, Mulugeta Melku, Zelalem Addis, Betelihem Terefe, Gashaw Yitayew

**Affiliations:** Department of Hematology & Immunohematology, School of Biomedical and Laboratory Sciences, College of Medicine and Health Sciences, University of Gondar, P.O. Box 196, Gondar, Ethiopia; Department of Immunology and Molecular Biology, School of Biomedical and Laboratory Sciences, College of Medicine and Health Sciences, University of Gondar, Gondar, Ethiopia; Department of Medical Microbiology, School of Biomedical and Laboratory Sciences, College of Medicine and Health Sciences, University of Gondar, Gondar, Ethiopia; Bahir Dar Regional Health Research Laboratory Center, Bahir Dar, Ethiopia

**Keywords:** Anemia, HIV, Risk factors, Children

## Abstract

**Background:**

Anemia is the most common hematological abnormalities in HIV patients and it is a wide spread public health problem. The World Health Organization estimates that over 2 billion people are anemic worldwide with more than 100 million of these anemic children living in Africa. In Ethiopia, there is limited information about the prevalence and factors associated with anemia among HIV positive children. Thus, this study aimed to determine the prevalence and associated factors of anemia among HIV infected children aged 6 months to 14 years in Gondar university Hospital antiretroviral treatment clinic.

**Methods:**

A cross-sectional study was conducted on 265 HIV infected children from February to June 2013 on HIV infected children attending Gondar university Hospital ART clinic. The study subjects were selected with systematic random sampling technique. Data of socio demographic characteristics and clinical conditions of the study subjects was collected using a structured pretested questionnaire. Hemoglobin value and CD4 counts were determined by cell Dyne 1800 and FACS count machine respectively. WHO Cut off value of hemoglobin was taken and adjusted to altitude to define anemia. Data was analyzed by using the SPSS version 20 statistical software and bivariate and multivariate logistic regression was used to identify predictors.

**Results:**

Anemia was present in 16.2 % (43 /265) of children, 60.5 % of them had mild anemia, 37.2 % had moderate anemia and 2.3 % had severe anemia. About 46.5 % of anemic children had normocytic-normochromic anemia followed by macrocytic-normochromic anemia (39.5 %). In this study, anemia was associated with eating green leafy vegetables (OR = 0.43, 95 % CI (0.188–0.981) and being on cotrimoxazole treatment (OR = 2.169, 95 % CI (1.047–4.49). But there was no significant association with age, sex, WHO clinical stage, opportunistic infections, intestinal parasitic infection and CD4 count percentage.

**Conclusions:**

The majority of HIV positive children in Northwest Ethiopia have a mild type of anemia and the increase in prevalence of anemia is due to being on cotrimoxazole and eating green leafy vegetables. Therefore, early diagnosis and treatment of anemia is essential in these patients.

## Background

Of HIV related hematological abnormalities, anemia is the most common hematological complication of HIV infection that has a significant impact on the quality of life and clinical outcomes [1]. Of patients, it is estimated that up to 90 % of adults and children develop anemia during HIV infection [2]. According to World Health Organization (WHO) definitions, anemia is defined as hemoglobin level less than 11 g/dl for children <5 years old, <11.5 g/dl for children 5–11.9 years old and <12 g/dl for children 12–14.9 years old after altitude adjustment [3–5]. Anemia is also classified as mild degree (Hgb 10.0–10.9 g/dl), moderate (Hgb 7.0–9.9 g/dl), severe (Hgb 4.0–7.0 g/dl), and very severe Hgb less than 4.0 g/dl. It can also be classified based on the Hematocrit (PCV) percent. Packed cell volume (PCV) of less than 33.0 % is regarded as anemia by the World Health Organization [3, 6, 7].

Anemia has been shown to be a significant predictor of progression to AIDS and several studies have shown that as hemoglobin levels decrease, the risk of HIV disease progression increases [8] and it is associated with an increased risk of death in both children and adult patients [1, 9–11]. It is a wide spread public health problem; The WHO estimates that over 2 billion people are anemic worldwide with more than 100 million of these anemic children living in Africa [12]. In East Africa, the prevalence of anemia ranges from 15–93 % [13]. According to the 2011 Ethiopia Demographic and Health Survey more than four in ten children (44 %) are anemic. One child of every five (21 %) has mild anemia, another 20 % have moderate anemia, and 3 % have severe anemia in the country [14]. But there is limited information about prevalence of anemia among HIV infected children in Ethiopia.

Anemia prevalence in children with HIV depends on several factors, such as stage of HIV infection, sex, age, race, and concurrent illness as well as the definition of anemia used. In general, as the HIV disease progresses, the prevalence and severity of anemia increases [9]. The high prevalence of anemia in HIV infected children in developing countries may be attributed to the fact that many of the children in these regions are also iron deficient which is compounded by poor socio-economic status [15, 16].

In addition to the direct effect of HIV, highly active antiretroviral therapy (HAART) also causes anemia. Although HAART has the capability of reducing the incidence of anemia [17] and lymphopenia by suppressing viral replication and increases CD4 cell count, anemia remains a common problem even for patients treated with antiretroviral agents. Anemia due to drugs, such as cotrimoxazole, pentamidine, foscarnet and zidovudine (AZT) often reflects reticuloendothelial iron block. Among the antiretroviral drugs, AZT is the most widely used drug that results in myelosuppression and thus anemia [18–21].

In Ethiopia, there is limited information about the prevalence and factors associated with anemia among HIV positive children. The study conducted in Jimma [18] is not enough to give conclusions to the general population. Since the normal hematological values are different in children on HAART and HAART naive, it is difficult to drive conclusions because the study conducted in Jimma only took those children who were on HAART. Therefore the current study determined the prevalence and associated factors of anemia among HIV infected children, aged 6 months to 14 years, in Gondar university Hospital ART Clinic.

## Methods

### Study setting and population

An institutional based, cross-sectional study was conducted from February to June, 2013 among 265 HIV infected children, aged 6 months to 14 years, attending Gondar university hospital. At the time of data collection there were 722 HIV infected children attending Gondar university hospital ART clinic. From them 265 were selected using systematic random sampling technique. A detailed history including, the socio-demographic characteristics, presenting symptoms, presence of symptoms related to anemia, nutritional history, past medical history, previous and current drug history, caretaker characteristics (including education level, occupation and income) was assessed with a pre-tested and a standardized questionnaire. All study subjects were approached during their respective appointments for follow up. After the interview, a detailed review of the medical records, such as concurrent opportunistic infections and WHO clinical staging of HIV disease was recorded. Then blood samples for hemoglobin determination and CD4 counts and stool samples for intestinal parasite were collected and processed.

### Laboratory analysis

About 4 ml of venous blood was collected by an experienced laboratory technologist from each subject for CD4 count and hematological parameters analysis. Hematological parameters; hemoglobin (Hgb), hematocrit (%), mean cell volume (MCV), mean cell hemoglobin (MCH), mean cell hemoglobin concentration (MCHC), red blood cell count (RBC) and red cell distribution width (RDW) were determined using the automated blood analyzer Cell-Dyne 1800 and CD4 count was assayed using the Becton Dickenson (BD) FACS caliber. For intestinal parasite examination a stool sample was collected and a drop of saline was mixed by an applicator stick on the slide and was examined for intestinal parasites by experienced laboratory technologist.

### Statistical analysis

The data was cleaned, edited, checked for completeness and entered in to the data program SPSS version 20 statistical software for analysis. Descriptive statistics were used to give a clear picture of dependent and independent variables. Bivariate and multivariate logistic regression was used to identify predictors.

### Ethical clearance

The study was conducted after ethical letters were obtained from university of Gondar Ethical Committee. Informed written consent was taken from the caretakers and in addition assent was obtained from older children (above 8 years) before enrollment in the study. Then the objective of this research was explained to the study participant’s, and those willing to participate were included. Participation in the study was voluntary and refusal was possible. To ensure confidentiality of data, study subjects were identified using codes and unauthorized persons were not able to access the collected data. The study participants’ result were reported to the physician for proper management.

## Results

### Socio-demographic and clinical characteristics of study participants

A total of 264 HIV positive children, aged between 6 months and 14 years old, participated in the study. One hundred thirty four were males and one hundred thirty one were females, M:F was almost 1:1 (50.6 and 49.4 % respectively). The mean age of the study participants was 9.3 ± 3.3 years. The biggest percentage (61.9 %) of children were aged 5 to 11.9 years. About 48.7 % were receiving cotrimoxazole prophylaxis and 70.9 % were on antiretroviral therapy (Table 1).

**Table 1:** Characteristics of HIV positive children attending Gondar university hospital ART clinic from February to June, 2013, Northwest Ethiopia

Characteristics		Frequency	Percentage
Age in years	0.5–4.9	24	9.1
	5–11.9	163	61.5
	12–14.9	78	29.4
Sex	Male	134	50.6
	Female	131	49.4
Residence	Urban	250	94.3
	Rural	15	5.7
Religion of guardian	Orthodox	233	87.92
	Muslim	29	10.94
	Protestant	3	1.13
Occupation of guardian	Merchant	47	17.7
	Farmer	23	8.7
	Government employed	49	18.5
	Non-employed	33	12.5
	Daily laborer	113	42.6
Educational status of guardian	Illiterate	78	29.4
	Elementary School	97	36.6
	High School	63	23.8
	Certificate and above	27	10.2
Meat and animal products intake	Yes	199	75.1
	No	66	24.9
Green leafy vegetables intake	Yes	219	82.6
	No	46	17.4
WHO stage	I	63	23.8
	II	72	27.2
	III	105	39.6
	IV	25	9.4
HAART	Yes	188	70.9
	No	77	29.1
Opportunistic infections	Yes	19	7.2
	No	246	92.8
Cotrimoxazole	Yes	129	48.7
	No	136	51.3

### Prevalence of anemia

**Fig. 1:**
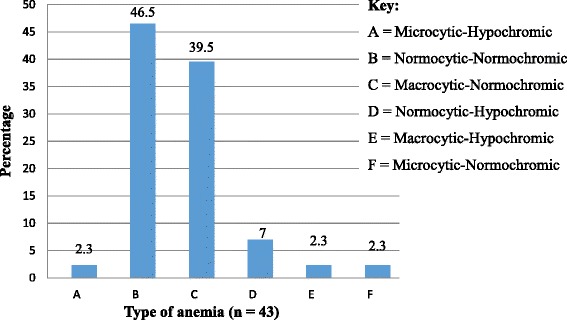
Type of anemia among HIV positive children attending Gondar University Hospital ART Clinic from February – June, 2013, Northwest Ethiopia

The prevalence of anemia among the study children was obtained by considering the cut off values of Hgb (<11 g/dl for children <5 years old, <11.5 g/dl for children 5–11.9 years old and <12 g/dl for children 12–14.9 years old) after altitude adjustments. The study area has an altitude of 2133 m and Hgb value was adjusted by subtracting 0.08 g/dl [3]). Based on this, prevalence of anemia was 16.2 % (43 /265). From the anemic children, 60.5 % of them had mild anemia, 37.2 % had moderate anemia and 2.3 % had severe anemia. Regarding the type of anemia, about 46.5 % of anemic children had normocytic-normochromic anemia followed by macrocytic-normochromic anemia (39.5 %) (Fig. 1).

To determine risk factors of anemia among HIV infected children, the bivariate and multivariate logistic regression was used to identify predictors. Based on this, anemia was associated with eating green leafy vegetables and taking cotrimoxazole treatment. But there was no significant association between anemia and socio-demographic characteristics, CD4 count, WHO clinical stage, parasitic infections and OPIs (Table 2).

**Table 2:** Association of socio-demographic and other clinical variables with anemia among HIV infected children attending Gondar university Hospital ART clinic from February – June, 2013

Variables		Anemic	Non-anemic	COR (95 % CI)	AOR (95 % CI)
Age	0.5–5years	4 (16.7 %)	20 (83.3 %)	1 (0.293–3.413)	1.388 (0.359–5.363)
	5–11years	26 (16.0 %)	137 (84.0 %)	1.054 (0.509–2.183)	1.285 (0.590–2.7980)
	11–14years	13 (16.7 %)	65 (83.3 %)	1^a^	1^a^
Sex	Male	20 (14.9 %)	114 (85.1 %)	1^a^	1^a^
	Female	23 (17.6 %)	108 (82.4 %)	0.824 (0.428–1.585)	0.741 (0.358–1.530)
Eating animal products	Yes	28 (14.1 %)	171 (85.9 %)	1^a^	
	No	15 (22.7 %)	51 (77.3 %)	0.557 (0.276–1.122)	
Eating green leafy vegetable	Yes	31 (14.2 %)	188 (85.8 %)	1^a^	1^a^
	No	12 (26.1 %)	34 (73.9 %)	0.467 (0.219–0.999)	**0.430 (0.188–0.981)**
WHO Clinical stage	I	15 (23.8 %)	48 (76.2 %)	1^a^	1^a^
	II	11 (15.3 %)	61 (84.7 %)	1.733 (0.73–4.116)	1.886 (0.758–4.689)
	III	16 (15.2 %)	89 (84.8 %)	1.738 (0.791–3.819)	1.497 (0.557–4.022)
	IV	1 (4.0 %)	24 (96.0 %)	7.5 (0.934–60.196)	4.806 (0.547–42.203)
HAART	Yes	24 (12.8 %)	164 (87.2 %)	1^a^	1^a^
	No	19 (24.7 %)	58 (75.3 %)	0.447 (0.228–0.875)	0.671 (0.272–1.652)
Opportunistic infections	Yes	6 (31.6 %)	13 (68.4 %)	0.384 (0.137–1.073)	0.416 (0.131–1.319)
	No	37 (15.0 %)	209 (85.0 %)	1^a^	1^a^
Cotrimoxazole	Yes	27 (20.9 %)	102 (79.1 %)	1^a^	1^a^
	No	16 (11.8 %)	120 (88.2 %)	1.985 (1.014–3.889)	**2.169 (1.047–4.490)**
Intestinal parasite infection	Yes	10 (15.4 %)	55 (84.6 %)	1.087 (0.503–2.348)	0.841 (0.373–1.895)
	No	33 (16.5 %)	167 (83.5 %)	1^a^	1^a^
CD4 percentage	<15 %	9 (18.8 %)	39 (81.2 %)	0.744 (0.306–1.81)	0.956 (0.341–2.686)
	15–25 %	17 (16.8 %)	84 (83.2 %)	0.848 (0.408–1.765)	1.022 (0.455–2.293)
	>25 %	17 (14.7 %)	99 (85.3 %)	1^a^	1^a^

^a^Reference category, bold numeric indicates statistically significant association

## Discussion

Anemia is a frequently encountered aberration in HIV patients [22] which may be clinically important. Multifactorial causes of anemia may complicate its original cause and/or its suitable treatment [23]. The pathogenesis of anemia in HIV infection is multifactorial which includes bleeding (gastrointestinal malignancy/severe infection), insufficient dietary intake (vitamins such as cobalamin and folate, iron, and general malnutrition), hemolytic anemia (i.e., malignancies, infections, splenomegaly, and immune dysfunction) and changes in erythropoietin synthesis and/or bone marrow suppression [2].

The prevalence of anemia in this study was 43/265 (16.2 %). This is lower than the reports of studies done in India [24], Cape Town [25], Uganda [11], Tanzania [26] and Jimma [18] which were 66, 73, 91.7, 21.9 and 44 %, respectively. On the other hand, it is higher than the study done in Northwest Brazil 13.6 % [27]. This difference may be due to differences in ethnicity, geographical location, study designs and time of study. Also there are age differences in the study participants, some took up to 12 years old children, some up to 10 years old children but the current study took children up to 14 years old.

In this study from anemic children, 60.5 % of them had mild anemia, 37.2 % moderate anemia and 2.3 % had severe anemia which is comparable with the study done in Uganda in 2002 [11] where they reported 35.1 % prevalence of moderate to severe anemia and from 2007 to 2009 [28] which showed 62.2 % patients had mild anemia and 32.0 % had moderate anemia while 4.8 % had severe anemia. In contrast, prevalence of severe life threatening anemia in our study, which was 2.3 %, was lower than study done in Jimma, which was 14.3 % [18].

In the current study about 46.5 % of anemic children had normocytic-normochromic anemia followed by macrocytic-normochromic anemia (39.5 %). But in Uganda [28] from 2007 to 2009 the predominant type of anemia was microcytic-hypochromic anemia (44.9 %) followed by normocytic-hypochromic anemia (26.5 %) and normocytic-normochromic anemia (19.0 %).

In this study, anemia was associated with eating green leafy vegetables and being on cotrimoxazole treatment. But there is no significant association with age, sex, residence, WHO clinical stage, HAART, opportunistic infections, intestinal parasitic infection and CD4 percentage. But in contradiction to this study, a study done in Tanzania showed that not being on HAART, having CD4 % <25 %, having a history of tuberculosis and having hookworm infestation, were independent risk factors for anemia [26]. A similar study on children aged 1 to 12 years in India, showed that age younger than 6 years old, rural residence, advanced HIV disease stage and TB infection were risk factors for anemia while HAART was protective while gender, cotrimoxazole and HAART regimen type had no association with anemia [24].

## Conclusion

Our findings showed that majority of HIV positive children in Northwest Ethiopia who are anemic have mild anemia and the prevalence of anemia is related to being on cotrimoxazole and eating leafy vegetables. Therefore, early diagnosis and treatment of anemia is essential in these patients. In this study direct wet mount was used for parasitic examination and this may less sensitive to detect ova of parasites. Also longitudinal studies on iron, folate, cobalamin levels and bone marrow examination, erythropoietin and viral load determination, are needed to determine the actual etiology of anemia in HIV infected children.

## Abbreviations

AIDS: Acquired immune deficiency syndrome
ART: Antiretroviral treatment
AZT: Zidovudine
CBC: Complete blood count
CD4^+^: Cluster differentiation 4
FACS: Florescence activated cell sorting
HAART: Highly active antiretroviral treatment
Hgb: Hemoglobin
HIV: Human immunodeficiency virus
MCH: Mean cell haemoglobin
MCHC: Mean cell haemoglobin concentration
MCV: Mean cell haemoglobin
OPI: Opportunistic infections
PCV: Packed cell volume
RDW: Red cell distribution width
TB: Tuberculosis
WHO: World Health Organization
